# Virtual reality guided focused Sylvian approach for clipping unruptured middle cerebral artery aneurysms

**DOI:** 10.3389/fsurg.2024.1411396

**Published:** 2024-07-01

**Authors:** Rui Zhang, Daniel Hänggi, Pia Köskemeier, Sajjad Muhammad

**Affiliations:** ^1^ Department of Neurosurgery, Medical Faculty, Heinrich-Heine-University, Dusseldorf, Germany; ^2^ Department of Neurosurgery, King Edward Medical University, Lahore, Pakistan; ^3^ Department of Neurosurgery, International Neuroscience Institute (INI), Hanover, Germany; ^4^ Department of Neurosurgery, University of Helsinki and Helsinki University Hospital, Helsinki, Finland

**Keywords:** 3-dimensional reconstruction, keyhole approach, middle cerebral artery (MCA) aneurysm, unruptured aneurysm, virtual reality

## Abstract

**Objective:**

The increasing prevalence of unruptured intracranial aneurysms, detected through advanced brain imaging, necessitates a cautious approach to surgical intervention, with a focus on minimizing associated risks. This retrospective study explores the safety and better aesthetic outcomes of a Virtual Reality (VR) guided Focused Sylvian Approach (FSA) in comparison to the standard Pterional Surgical Approach (SPA) for the clipping of unruptured small-medium-size (<10 mm) Middle Cerebral Artery (MCA) aneurysms.

**Methods:**

23 patients with 23 unruptured MCA aneurysms underwent the VR-guided FSA from June 2020 to September 2023, while 22 patients with 23 unruptured MCA aneurysms who underwent SPA were retrospectively recruited from the medical records database from January 2017 to May 2020. The comparative analysis involved surgical duration, postoperative complications, hospital stay, and a three-month follow-up patient's sequela survey.

**Results:**

All aneurysms were effectively treated. The FSA procedure demonstrated a shorter surgical duration compared to the SPA group (164 ± 48 min vs. 196 ± 133 min, *P* = 0.2974). Despite a slightly higher median age in the FSA group (59 vs. 56 years), the median hospital stay was shorter in the FSA group (6 days) compared to the SPA group (7 days). The SPA group exhibited a higher incidence of complications (17/23) including cephalalgia, scar irritation, scar numbness, and temporal muscle dysfunction, compared to the FSA group (1/23), with a statistical significance of *P* < 0.05. Although FSA cannot demonstrate significant surgical efficiency in surgical duration and hospitalization, its superior aesthetics and preservation of temporalis muscle function compared to the SPA group.

**Conclusion:**

The VR-guided FSA offers improved aesthetics and preservation of muscle function compared to the SPA. Our retrospective study underscores the potential benefits of VR-guided, personalized, focused Sylvian approaches for managing unruptured small-medium-size MCA aneurysms.

## 1 Introduction

The standard pterional approach, as delineated by Yasargil (1), has traditionally been employed for the clipping of aneurysms situated on the anterior aspect of the circle of Willis. Nevertheless, the conventional pterional approach, characterized by a sizable bone flap and extensive opening of the Sylvian fissure, may precipitate iatrogenic complications, encompassing brain contusion, venous infarction, subdural effusion, and seizures (2, 3).

In an effort to mitigate postoperative complications associated with the standard pterional approach, several more tailored approaches have been introduced and effectively implemented in clinical practice. These approaches include the supraorbital, lateral supraorbital (LSO), and mini-pterional approaches as described by various authors (4–7). Elsharkawy et al. introduced the focused opening of the Sylvian fissure in 2014 specifically for the clipping of MCA aneurysms (8). Despite their modifications, all of these approaches still maintain their focus on the sphenoid ridge as a critical anatomical landmark and the skin incision and bone flap remain relatively large.

This study presents the feasibility of utilizing a VR-guided FSA for clipping incidental small-medium-sized MCA aneurysms. This approach involves the use of VR techniques and 3-dimensional (3D) reconstruction image assessment, a dynamic and precise burr hole, minimal craniotomy, and limited opening of the Sylvian fissure. By employing this technique, we observed swift recovery in our patients avoiding potential perioperative complications associated with more invasive approaches. The FSA demonstrates promise as a safe and optimized option for treating incidental medium-sized MCA aneurysms.

## 2 Materials and methods

### 2.1 Patient population and characteristics

The inclusion criteria for this study comprised patients diagnosed with unruptured small to medium-sized MCA aneurysms (size <10 mm) necessitating craniotomy for clipping. Additionally, the availability of detailed medical records during the perioperative period and a follow-up period of 3 months were necessary for inclusion. Patients with multiple aneurysms (three or more) and a history of subarachnoid hemorrhage were excluded from the study.

Between January 2017 and September 2023, a total of 46 surgical procedures were performed on 45 patients in our department. Detailed data for each group can be found in Table 1, providing comprehensive information about the patients and their respective surgical approaches.

**Table 1:** Patient demographics, aneurysm localization, surgical duration, and hospitalization period in groups.

Group	FSA	SPA	*P*-value
Patient	*n* = 23	*n* = 22^#^	
Operation	*n* = 23	*n* = 23^#^	
Age (year)	Welch's test		*P = 0.3785*
Mean ± SD	57.87 ± 10.19	55.22 ± 10.03	
Min–Max	35–77	35–70	
Median	59	56	
Gender	Fisher's exact test		*P = 0.3368*
Male	5	8	
Female	18	14	
Aneurysm location	Fisher's exact test		*P = 0.3726*
Right	12	8	
Left	11	15	
Surgical duration (minutes)	Welch's test		*P = 0.2974*
Mean ± SD	164 ± 48	196 ± 133	
Min–Max	120–360	120–780	
Median	150	180	
Hospitalization period (day)	Welch's test		*P = 0.1525*
Mean ± SD	5.87 ± 2.26	7.13 ± 3.48	
Min–Max	3–13	3–18	
Median	6	7	

SPA, standard pterional approach; FSA, focused Sylvian approach.

^#^One patient from SPA group had bilateral MCA aneurysms underwent two separated surgery.

### 2.2 Image process and preoperative planning

**Figure 1:**
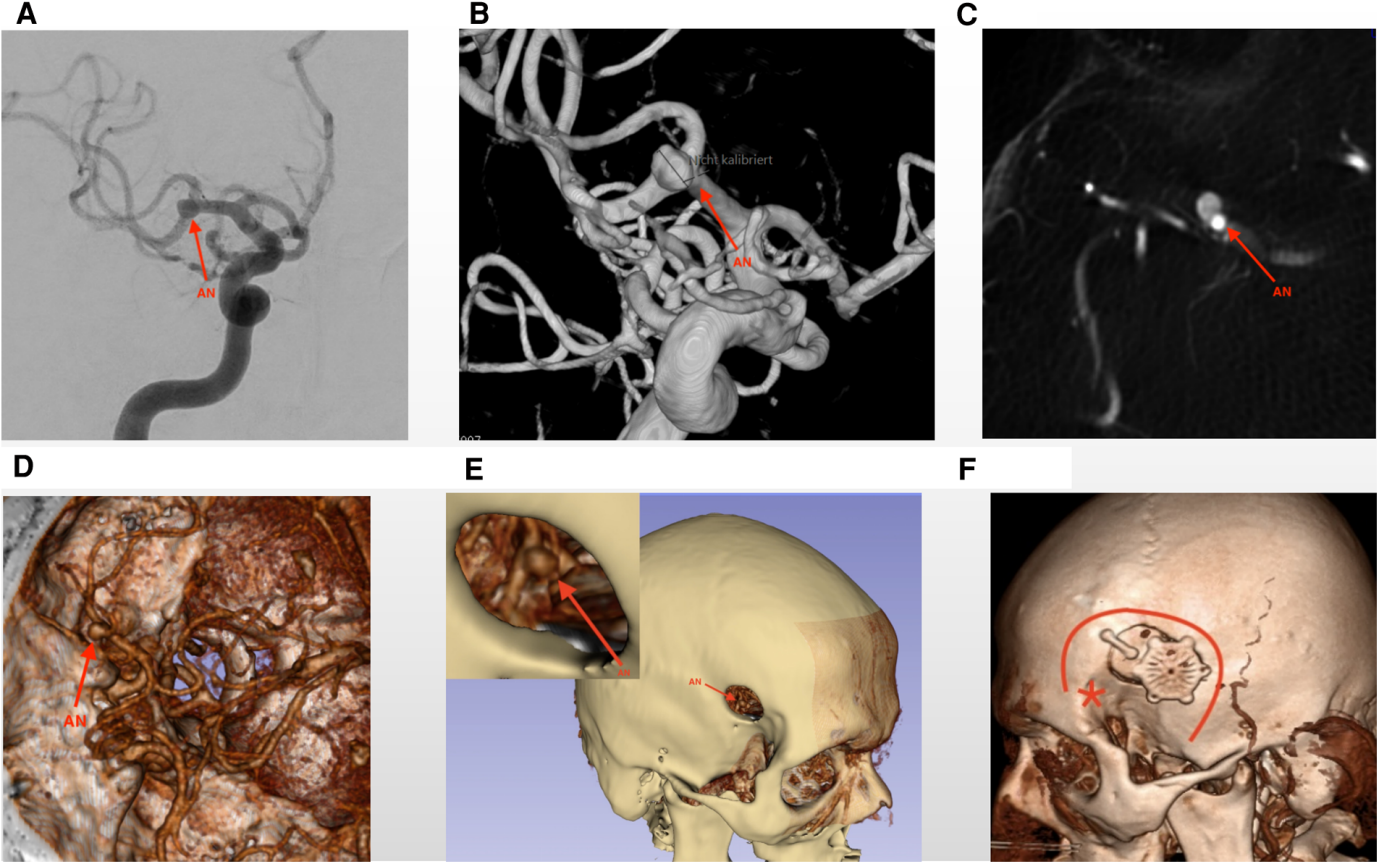
Virtual reality assessment of the preoperative images. The figures illustrate preoperative images depicting a growing aneurysm located at the MCA bifurcation. Sub-figures (**A–C**) showcase traditional DSA (Digital Subtraction Angiography) and CTA (Computed Tomography Angiography) images, providing initial insights into the aneurysm. (**D**) Presents 3D reconstruction images, offering enhanced visualization of surrounding anatomical structures and indicating the direction of the aneurysm dome. (**E**) Presents a virtual reality imitation operation picture, which includes a local magnification that highlights the bone window. Through this window, the aneurysm becomes visible, aiding in surgical planning and visualization. (**F**) The postoperative image illustrates the bone flap and the dimensions of the bone window of the Focused Sylvian Approach (FSA). The red asterisk (*) denotes the key burr hole utilized in the Standard Pterional Approach (SPA), while the red curved line delineates the extent of the craniotomy for the SPA approach. The FSA bone window is notably smaller than that of the SPA. The red arrows in Figure 1 indicate to the unruptured MCA aneurysm. AN, aneurysm.

Integrating VR techniques and 3D reconstruction images has proven to be highly valuable in our preoperative planning process (9, 10). To accomplish this, we leverage computed tomography angiography (CTA) data and utilize an open-source computer software known as 3D Slicer (www.3dslicer.org) to convert conventional images into 3D representations preoperatively. This method facilitates a more comprehensive visualization of the patient's anatomy and the specific details of the aneurysm. The 3D reconstruction images obtained through this process assist in accurately assessing the aneurysm's morphology, its location within the Sylvian fissure, and the relationship between the aneurysm and surrounding structures. The incorporation of VR and 3D reconstruction images into our preoperative planning enhances our understanding of the patient's unique anatomy, thereby enabling us to formulate a more precise and tailored surgical plan for clipping the MCA aneurysm (Figure 1).

### 2.3 Surgical procedure

**Figure 2:**
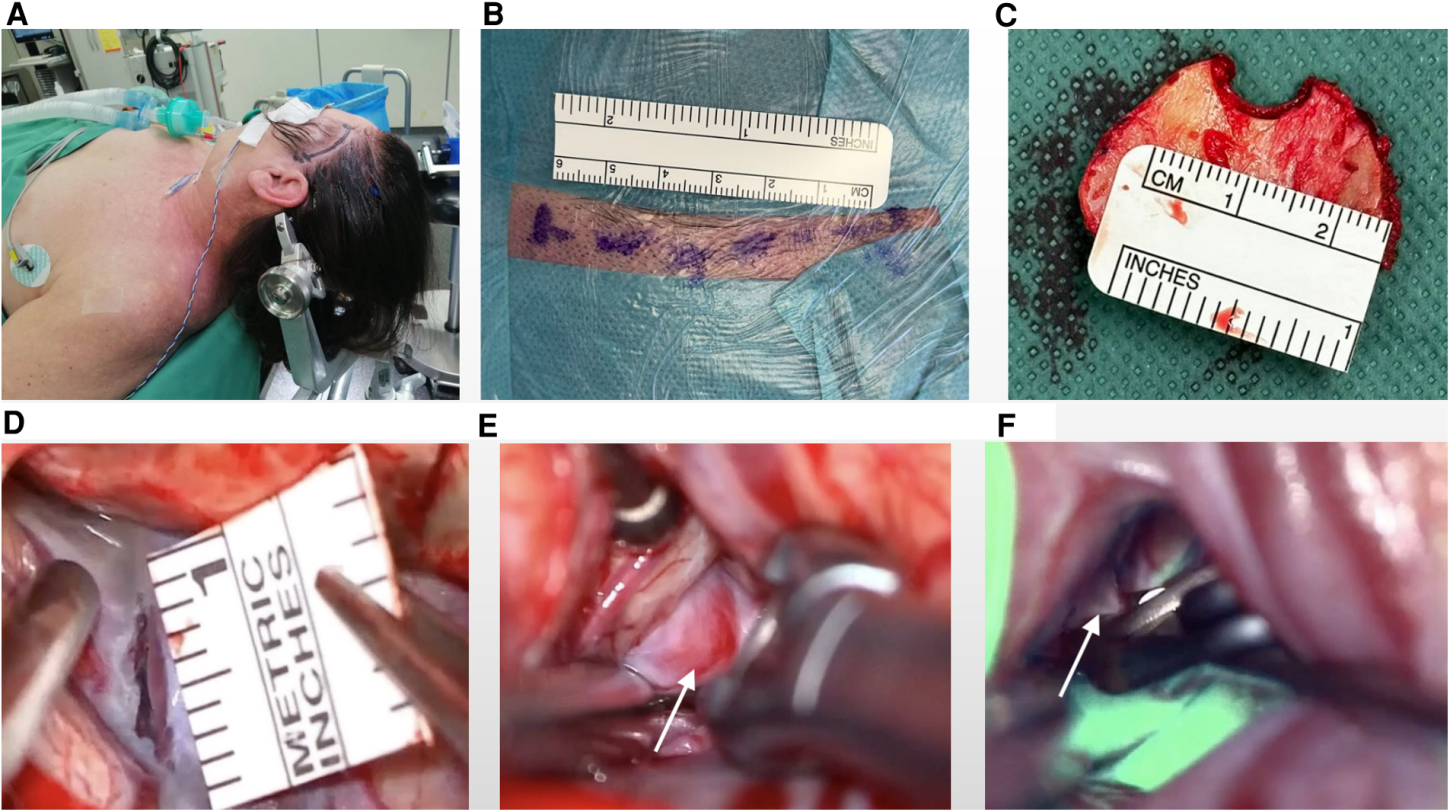
Surgical positioning of patients and intraoperative visualizations. (**A**) Optimal patient positioning for procedural precision. (**B**) Marking of scalp incision (approximately 6 cm). (**C**) Identification of a marked 2 cm diameter bone flap to expose the surgical field. (**D**) Dissection extent of the Sylvian fissure for access, measuring approximately 1 cm in length. (**E**) Visual representation of the clipping procedure emphasizing the aneurysm body (indicated by white arrow). (**F**) Illustration of the clipping procedure delineating the aneurysm neck (indicated by white arrow) and demonstrating parent vessel patency and no perfusion in aneurysm using ICG (Indocyanine Green).

The patient was positioned supine with the head elevated and fixed using a Mayfield head fixation device. A slightly curved incision was made in the scalp from the pre-auricular to the superior temporal line. A small incision was made on the temporalis muscle with the shape “L”: a cutting along the muscle fiber then 90° turning under the superior temporal line. The burr hole position was determined based on preoperative imaging. A 2.5 cm diameter bone flap was created that was focused on the Sylvian fissure. The Sylvian fissure opening was based on the location of an aneurysm in the Sylvian fissure following preoperative 3D CTA analysis. The dura was opened in a curved shape, and the arachnoid membrane was incised sharply. The Sylvian fissure was carefully dissected using sharp and blunt techniques (11). The length of the fissure opening varied depending on the aneurysm's size and location. Temporary clipping was used when necessary, and a permanent clip was applied once the aneurysm was fully exposed. Indocyanine green (ICG) angiography, intraoperative neuromonitoring (IONM), and Doppler ultrasonography were consistently used for all procedures. In some cases, the aneurysm was resected for research purposes (Figure 2, Supplementary Video S1).

### 2.4 Surgical duration, postoperative complications, hospital stay, and follow-up

The duration of the surgery was quantified from the initial skin incision to the final skin closure (“skin-to-skin” time). Acute postoperative complications and the length of hospital stay were documented from hospital records. Long-term complications were evaluated at a 3-months follow-up visit using a standardized survey form. An independent physician assessed the outcomes, and all surgical procedures were conducted by the senior author.

### 2.5 Statistical analysis

Data analysis was performed using statistical software (R, Version 3.6). Surgical duration, patients' age, and hospitalization period were recorded with maximum, minimum, median, and mean ± standard deviation (SD). Those data were compared in groups with Welch's Test. Patients' gender, aneurysm locations, and sequela were descriptively recorded and compared with Fisher's exact test. All statistical tests with a *P*-value <0.05 were considered statistically significant.

## 3 Results

### 3.1 Included patients characteristics

The FSA group consisted of 23 patients, including 5 males and 18 females. Among them, 12 patients had right-side aneurysms and 11 had left-side aneurysms. The age in this group was from 35 to 77 years, with a median age of 59 years.

In comparison, the SPA group comprised 22 patients, with 8 males and 14 females. Among them, 1 patient had bilateral aneurysms, 7 patients had right-side aneurysms and 14 had left-side aneurysms. The age range was also 35–70 years, with a median age of 56 years.

**Figure 3:**
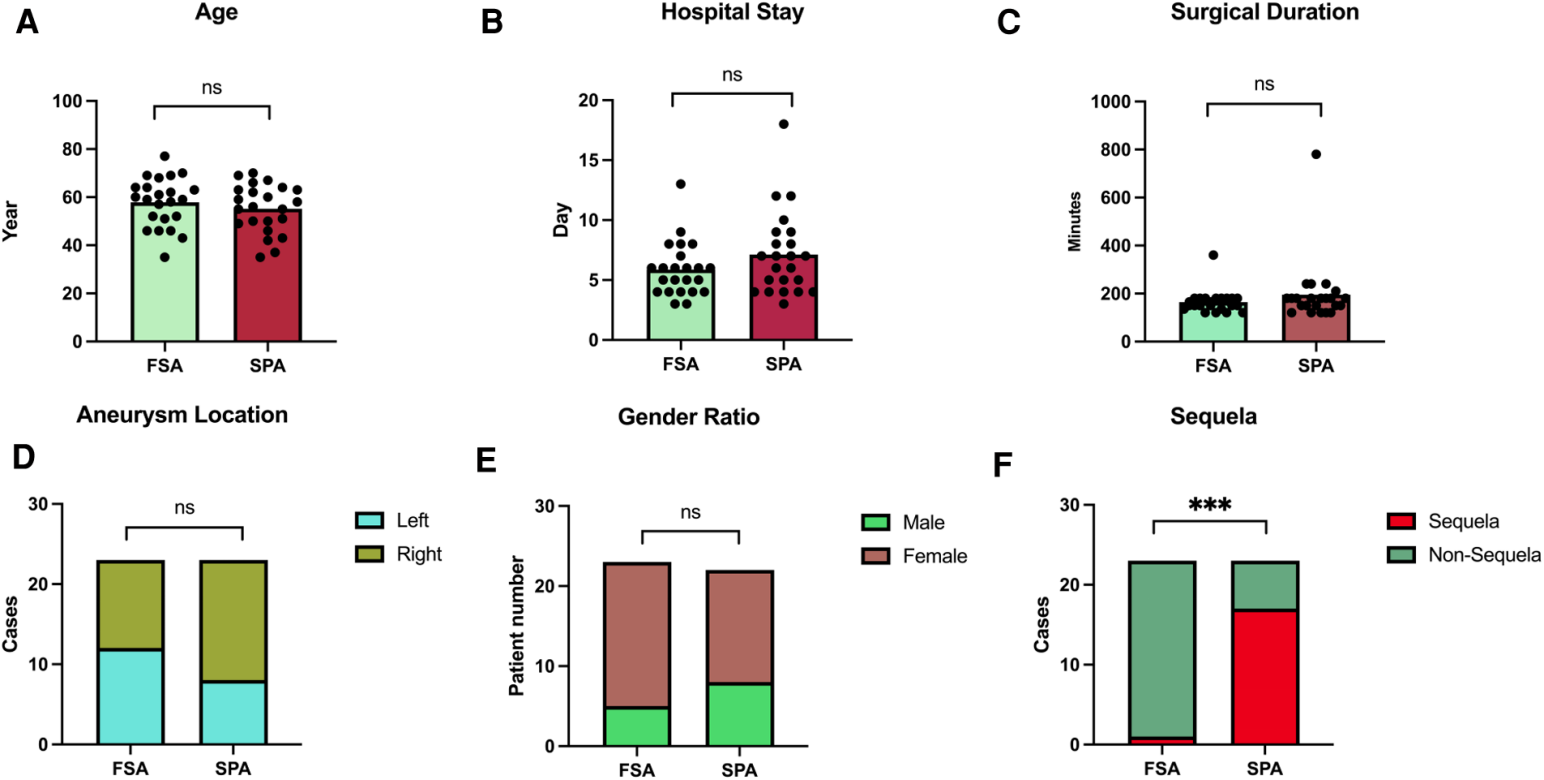
Statistical analysis of focused Sylvian approach (FSA) group and standard pterional approach (SPA) group. (**A–E**) Depict the characteristics of patients from the FSA and SPA groups, revealing no significant differences in age, hospital stay, surgical duration, aneurysm locations, and gender ratio. (**F**) Illustrates a significant bias between FSA and SPA groups in terms of 3-month follow-up sequelae, with a *p*-value <0.05.

Both groups were similar in terms of demographic and clinical characteristics with a *P*-value >0.05 (Table 1 and Figure 3).

### 3.2 Surgical duration and hospitalization period

The median surgical duration in the FSA group was 150 min (120–360 min); while for the SPA group, it was in average 180 min (120–780 min). The median hospitalization stays for patients in the FSA group was 6 days (3–13 days), while for the SPA group, it was 7 days (3–18 days).

There was no significant difference in the surgical duration and hospitalization days between the two groups, *P*-value >0.05 (Table 1, Figure 3).

### 3.3 Perioperative complications, radiological and clinical outcome, and 3-month follow-up

At the 3-month follow-up CT angiography, complete aneurysm obliteration was observed in all patients from both the FSA and SPA groups. However, notable differences were observed in the incidence of long term sequela between the two groups. Specifically, within the SPA group, one patient experienced transient aphasia post-operatively, which was recovered by the 3-month follow-up. A substantial proportion of patients (17 out of 23, 73.91%) in the SPA group reported various subjective or objective deficits and sequelae, including cephalalgia, scar area numbness, scar area sensory disturbance, scar irritation, wound healing issues, chewing pain, and chewing weakness at the time of the survey.

In contrast, within the FSA group, one patient developed temporary right leg hemiparesis, which completely resolved during the hospital stay, and one patient continued to experience cephalalgia at the survey. A Fisher's exact test comparing the 3-month follow-up outcomes between the groups yielded a statistically significant with *P*-value <0.05. Then the preservation of scalp and temporal muscle function was notably superior in the FSA group compared to the SPA group (Table 2, Figure 3).

**Table 2:** Postoperative complications at three-month follow-up.

Sequela	FSA	SPA
Cephalalgia	1	5
Numbness(scar area)	0	4
Sensory disturbance	0	3
Scar irritating	0	2
Chew pain	0	1
Chew weakness	0	1
Wound healing defect	0	1

FSA, focused Sylvian approach; SPA, standard pterional approach. Compare the sequela from FSA and SPA groups with Fisher's exact test, *P* < 0.0001.

## 4 Discussion

Intracranial saccular or berry-like aneurysms are relatively common, occurring in approximately 3% of the population. The detection of unruptured intracranial aneurysms has increased due to the widespread use of cross-sectional imaging techniques in clinical practice (12). Currently, there is no single tool to accurately detect rupture-prone intracranial aneurysms leaving the possibility of over-treatment. Thus the risk of treatment for unruptured aneurysms should be minimized. However, there is still ongoing debate and varying opinions regarding the optimal treatment approach for unruptured aneurysms, particularly in the case of unruptured MCA aneurysms (13, 14). It is crucial to carefully consider several factors before deciding on treatment, including the shape, size, location, and growth of the aneurysm, the patient's medical and family history, smoking, hypertension, excess alcohol consumption, psychological stress of the patient and the available interventional/surgical experience. If multiple risk factors are present and treatment risk is low then, we believe that treatment should be considered for patients with unruptured aneurysms (12).

Certainly, endovascular coiling presents a comparatively lower degree of invasiveness when compared to surgical clipping in the therapeutic management of intracranial aneurysms. Nevertheless, the technique of clipping continues to manifest distinct merits, particularly in instances involving aneurysms characterized by wide-neck morphology or with complicated perforators. The determination of the most appropriate therapeutic approach has been the subject of deliberation within numerous comprehensive prospective randomized clinical trials, exemplified by studies such as the International Subarachnoid Aneurysm Trial (ISAT) (15) and the Barrow Ruptured Aneurysm Trial (BRAT) (16). Notably, surgical intervention remains the primary therapeutic preference for aneurysms situated along the middle cerebral artery.

Additionally, when considering economic factors, surgical clipping is associated with relatively lower costs compared to endovascular coiling (17, 18). Therefore the craniotomy strategy needs to be consistently improved to keep the risk of treatment as low as possible to justify treatment.

The pterional approach, initially described by Hamby in 1964 and further developed by Yasargil in the 1970s, has been the standard surgical window for clipping anterior Willis circle aneurysms (1, 19). The introduction of the surgical microscope significantly improved outcomes for patients with aneurysms. Despite continuous improvements in the approach by generations of neurosurgeons, postoperative complications still occur occasionally after pterional craniotomy. These complications include brain contusion, venous infarction, subdural collections, seizures, scar irritation, scar area sensitivity, and even temporalis muscle atrophy (20). The larger wound area and greater dissection of muscle are likely the main causes of these complaints.

In this study, we demonstrate the safety and feasibility of a highly focused and minimally invasive Sylvian approach for clipping small-medium-sized unruptured MCA aneurysms. This tailored approach has shown faster recovery and shorter hospital stay. Our goal was also to reduce the size of the skin incision and incision in the temporal muscle to overcome scar irritation and temporal muscle atrophy. Similarly, minimizing the size of the craniotomy and adopting a minimally invasive approach to open the Sylvian fissure may reduce the risk of iatrogenic injury while maintaining the quality of the surgical procedure. The length of the Sylvian fissure opening can vary depending on the size and location of the aneurysm. Previous research articles have suggested a minimum length of around 10–15 mm for the opening (8). However, in our practical work, we have found that a 10 mm dissection of the Sylvian fissure is sufficient for the exchange of instruments and the clipping process. This is achieved through detailed preoperative evaluation and optimal selection of the approaching angle. With the assistance of cutting-edge image processing technology and surgical experience, we propose that the modified focused Sylvian approach reduced the area of surgical field exposure and minimizes iatrogenic side effects. The VR-guided FSA offers improved aesthetics and preservation of muscle function compared to the SPA. Our retrospective study underscores the potential benefits of VR-guided, personalized, focused Sylvian approaches for managing unruptured small-medium-size MCA aneurysms.

## 5 Specific considerations

1. The position of the craniotomy on the Sylvian line in relation to the aneurysm dome and the determination of the route from the cortex to the aneurysm is critical. Preoperative planning using virtual reality image processing is beneficial in achieving these goals. The general principle is to avoid encountering the dome, which can obstruct the visibility of the neck of the aneurysm through the narrow opening of the Sylvian fissure.
2. A relaxed brain is essential for successful surgery, particularly in a narrow surgical corridor. By releasing cerebrospinal fluid in the cistern of the Sylvian fissure, a slack brain can be easily obtained. Opening the cistern early in the procedure is recommended.
3. Temporary clipping is not necessary as long as the dome of the aneurysm is radiographically smooth and rounded. We partially expose the proximal parent vessel but avoid temporary clipping, as each additional surgical step may pose a risk.
4. Crisis assessment and management are fundamental during the operation. Patient safety should not be compromised in the pursuit of minimally invasive surgery. The greatest risk is the unanticipated rupture of the aneurysm, which did not occur in this group of patients, likely due to the small number of cases. With our limited experience, this approach appears to be safe in our hands.

## 6 Limitations

It is important to note that this approach has limitations. It is not suitable for larger or complex MCA aneurysms, particularly ruptured aneurysms. Proficiency in microsurgical skills and experience in working through a narrow surgical corridor is essential for the clinical application of the focused Sylvian approach.

This study was retrospective single-centre research, sharing an inherent limitation with a narrow spectrum of surgeons and a small number of patients. Further multicenter research should be performed in the future.

## Data Availability

The original contributions presented in the study are included in the article/**Supplementary Material**, further inquiries can be directed to the corresponding author.

## Supplementary material

Supplementary Video S1 Surgical procedure of focused Sylvian approach. Link of video: https://youtu.be/i73Q6-lAg_o.
